# Amygdala-targeted neurofeedback for dissociative symptoms in PTSD: Converging evidence from three independent studies

**DOI:** 10.1016/j.psychres.2025.116752

**Published:** 2025-09-28

**Authors:** Tal Harmelech, Talma Hendler, Guy Gurevitch, Naomi Fine, Eyal Fruchter, Daniela Amital, Nadav Goldental, Raz Gross, Matthew A Robinson, Lauren AM Lebois, Milissa Kaufman, Aron Tendler

**Affiliations:** a GrayMatters Health, Haifa, Israel; b Sagol Brain Institute, Tel Aviv Sourasky Medical Center, Tel Aviv, Israel; c Gray Faculty of Medical & Health Sciences, Tel Aviv University, Tel Aviv, Israel; d School of Psychological Sciences, Faculty of Social Sciences, Tel Aviv University, Tel Aviv, Israel; e Sagol School of Neuroscience, Tel Aviv University, Tel Aviv, Israel; f ICAR Collective and Brus Rappaport Medical Facility of the Technicon, Haifa, Israel; g Barzilai Medical Center, Ashkelon, Israel; h Faculty of Health Sciences, Ben-Gurion University, Beer Sheva, Israel; i Sheba Medical Center, Ramat Gan, Israel; j Division of Depression and Anxiety Disorders, McLean Hospital, Belmont, Mass, USA; k Department of Psychiatry, Harvard Medical School, Boston, Mass, USA

**Keywords:** PTSD, Dissociation, EFP neurofeedback, Amygdala, Depersonalization, Derealization

## Abstract

**Background:**

Dissociative symptoms occur in 14–30% of PTSD cases and present significant treatment challenges. These symptoms may respond to amygdala-targeted neural regulation training.

**Methods:**

Secondary analysis of patients with clinically significant dissociative symptoms (CAPS-5 Q29/Q30 >1) from three independent studies: Study 1 - comparison of amygdala neurofeedback + treatment-as-usual (n=14) vs. treatment-as-usual alone (n=7); Study 2 - single-arm neurofeedback study (n=14); Study 3 - comparison of amygdala neurofeedback (n=5) vs. control (n=2).

**Results:**

Large significant within-group improvements in both primary neurofeedback studies (Fine: d=1.82 p<0.001; Fruchter: d=0.85, p=0.007) contrasted with small non-significant effects in control groups. Mixed-effects analysis across all studies showed significant time effects (F=19.8, p<0.001) but non-significant Group × Time interactions. Specificity analysis revealed negligible correlations between dissociation improvements and other PTSD symptom changes (all r<0.17), indicating dissociation-specific rather than general therapeutic effects.

**Conclusions:**

Convergent evidence from independent studies supports preliminary efficacy of amygdala-targeted neurofeedback for dissociative symptoms. Larger controlled trials with standardized protocols are warranted.

## 1. Introduction

### 1.1. Dissociative PTSD: clinical significance and treatment challenges

Dissociative symptoms occur in approximately 14–30 % of individuals with PTSD and represent a distinct clinical phenotype with significant implications for treatment planning and outcomes (Lanius et al., 2010; Wolf et al., 2012, 2012; Stein et al., 2013). The clinical presentation of dissociative PTSD is characterized by prominent depersonalization and derealization symptoms (American Psychiatric Association, 2013). These symptoms manifest as feelings of detachment from oneself (e.g., out of body experiences, feeling as if one doesn’t have a body, disturbances in the sense of personal identity) or detachment from the environment (altered perceptions of space, feeling as if one is in a movie). These presentations reflect broader patterns of trauma-related affect dysregulation and adaptation (Wolf et al., 2012). While dissociative symptoms can impact one’s continuity of experience, it is theorized that they serve adaptive functions in the context of overwhelming emotional states, providing temporary relief from intolerable feelings at the cost of integrated conscious experience (Putnam, 1991; Spiegel et al., 2013).

Treatment challenges in the dissociative subtype of PTSD are multifaceted (Lanius et al., 2010). Traditional exposure-based therapies may be poorly tolerated or ineffective when dissociative symptoms interfere with emotional processing and integration (Lanius et al., 2010; Wolf et al., 2012; Stein et al., 2013) Dissociative states can impair emotional amygdala-related learning and fear extinction (Ebner-Priemer et al., 2009; Kleindienst et al., 2016) and subsequently impede the formation of coherent trauma narratives necessary for many evidence-based interventions (Resick et al., 2012; Cloitre et al., 2012; Price et al., 2014; Wolf et al., 2016; Bae et al., 2016). Additionally, dissociative symptoms themselves often become a source of distress and functional impairment that requires targeted intervention (American Psychiatric Association, 2013). Traumatized individuals with dissociative symptoms typically have co-occurring psychiatric conditions (Brand et al., 2009), high rates of self-destructive behaviors and suicidality (Foote et al., 2006), and are disproportionate treatment utilizers (Macy, 2002; Mansfield et al., 2010).

### 1.2. Neurobiology of dissociative PTSD

The DSM-5 recognition of a dissociative subtype of PTSD, in part, reflects growing evidence for distinct neurobiological profiles underlying these presentations (American Psychiatric Association, 2013; Lanius et al., 2012; Wolf et al., 2012). These findings align with broader recognition of complex presentations of PTSD, including proposals for complex PTSD in the ICD-11 classification system (Cloitre et al., 2013). Patients with dissociative PTSD demonstrate altered patterns of neural connectivity and activation that differ from those seen in classic hyperarousal-predominant PTSD (Lanius et al., 2010; Nicholson et al., 2017).

Consistent with theories that suggest dissociative symptoms are a type of emotion/arousal over-regulation, foundational neurobiological work indicates that those with the dissociative subtype of PTSD typically exhibit an excess of corticolimbic inhibition in response to emotionally provocative stimuli (Lanius et al., 2018). Specifically, cortical regions (e.g., ventromedial prefrontal cortex) are hyperactive, whereas limbic regions (e.g., amygdala) are hypoactive in these contexts compared to individuals with classic hyperarousal-predominant PTSD (Lanius et al., 2018; Lebois et al., 2022), see also the related reciprocal inhibition model (Chiba et al., 2021). These findings suggest when regulatory systems become imbalanced, the normal dynamic interplay between bottom-up emotional signals and top-down cognitive control can be disrupted, leading to either overwhelming emotional activation or excessive suppression of emotional and sensory experience. Dissociative symptoms represent the latter pattern, where overactive regulatory control leads to disconnection from typical emotional and bodily awareness.

Moreover, neuroimaging work implicates altered large-scale neurocognitive brain network connectivity in the dissociative subtype of PTSD that is distinct from classic PTSD (Lanius et al., 2018; Lebois et al., 2022a; Roydeva and Reinders, 2021; Lotfinia et al., 2020; Nicholson et al., 2020; Lebois et al., 2021; Lebois et al., 2022b). These findings correspond to network dysfunction models that propose psychopathology arises from disrupted connectivity and coordination between large-scale brain networks involved in self-generated thought (default mode network), executive control (central executive network), and salience detection (salience network) (Menon, 2015). These models have moved beyond simple “up-regulation” or “down-regulation” models to emphasize regulatory flexibility as a key component of healthy brain function (Menon, 2015; Ros et al., 2013). From this perspective, dissociative symptoms reflect reduced flexibility in neural regulation, with individuals becoming “stuck” in particular regulatory states rather than adapting dynamically to changing environmental and internal demands. This framework suggests that effective interventions should target the restoration of regulatory flexibility rather than simply increasing or decreasing activation in specific brain regions.

### 1.3. Amygdala as a therapeutic target for dissociative symptoms

The amygdala represents a promising therapeutic target for dissociative symptoms based on its central role in limbic and salience networks (Menon, 2015; Cunningham and Brosch, 2012), and the prior research implicating the amygdala and the limbic and salience networks in dissociative symptoms. Rather than viewing the amygdala simply as an alarm system that needs to be “turned down,” contemporary models conceptualize it as a flexible regulatory hub that coordinates responses across multiple neural networks based on contextual demands (Menon, 2015; Cunningham and Brosch, 2012). Its position as a hub connecting emotional processing, attention, and regulatory control systems makes it well-suited for interventions aimed at restoring balanced network function. This perspective builds on extensive research demonstrating the amygdala’s role not only in threat detection and fear responses but also in salience attribution, attention allocation, and the coordination of physiological and behavioral responses (Menon, 2015; Cunningham and Brosch, 2012). The amygdala’s extensive connectivity to cortical and subcortical regions positions it as a central node in the networks that support integrated conscious experience and self-awareness.

The amygdala’s involvement in interoception and self-processing further supports its relevance to dissociative symptoms, which fundamentally involve disruptions in self-awareness and embodied experience (Cunningham and Brosch, 2012; Craig, 2009). Moreover, amygdala complex connectivity maps can accurately classify the dissociative subtype of PTSD, classic PTSD, and nonpsychiatric control participants in recent machine learning work (85 % balanced accuracy, *p* < 0.001) (Nicholson et al., 2019). Together, this evidence suggests the amygdala in particular may be a fruitful target in neurofeedback interventions for dissociative PTSD.

### 1.4. Amygdala-targeted neurofeedback

Recent advances in real-time neuroimaging have enabled the development of neurofeedback protocols that can target specific brain regions, including deep structures like the amygdala, with high precision (Ros et al., 2013; Keynan et al., 2016). EEG-based approaches, while lacking the spatial resolution of fMRI, offer several advantages for clinical application, including lower cost, greater accessibility, and reduced burden on patients (Keynan et al., 2016). The development of EEG-fMRI fusion protocols has enabled the translation of fMRI-based targeting approaches to EEG platforms, making neurofeedback more scalable for clinical implementation (Keynan et al., 2016; Fruchtman-Steinbok et al., 2021).

Amygdala-targeted neurofeedback represents a novel approach that aims to restore regulatory flexibility rather than simply increasing or decreasing amygdala activation (Fine et al., 2024; Fruchter et al., 2024; Fruchtman-Steinbok et al., 2021). By providing real-time feedback about amygdala activity, these protocols may help individuals develop greater awareness and control over their regulatory states, potentially breaking the rigid patterns that characterize dissociative symptoms.

### 1.5. Study rationale and hypotheses

The current analysis examines consistency of findings across three independent studies that investigated amygdala-targeted neurofeedback in patients with PTSD and clinically significant dissociative symptoms. This approach allows for evaluation of consistency across different research studies, populations, and protocol variations while acknowledging the methodological heterogeneity that limits direct pooling of results.

The primary research question addressed in this analysis is whether amygdala-targeted neurofeedback demonstrates superior outcomes for dissociative symptoms compared to control conditions. Secondary questions include whether effects are sustained over time and whether similar patterns of improvement are observed across different dissociative symptom domains (depersonalization and derealization).

Given the theoretical framework and preliminary evidence from individual studies, we hypothesized that amygdala-targeted neurofeedback would show consistent beneficial effects across studies, with effect sizes that are clinically meaningful even in the context of methodological heterogeneity. We further hypothesized that these effects would be evident for both depersonalization and derealization symptoms, consistent with shared underlying mechanisms involving amygdala-mediated regulatory networks.

## 2. Methods

### 2.1. Study overview

**Fig. 1.**
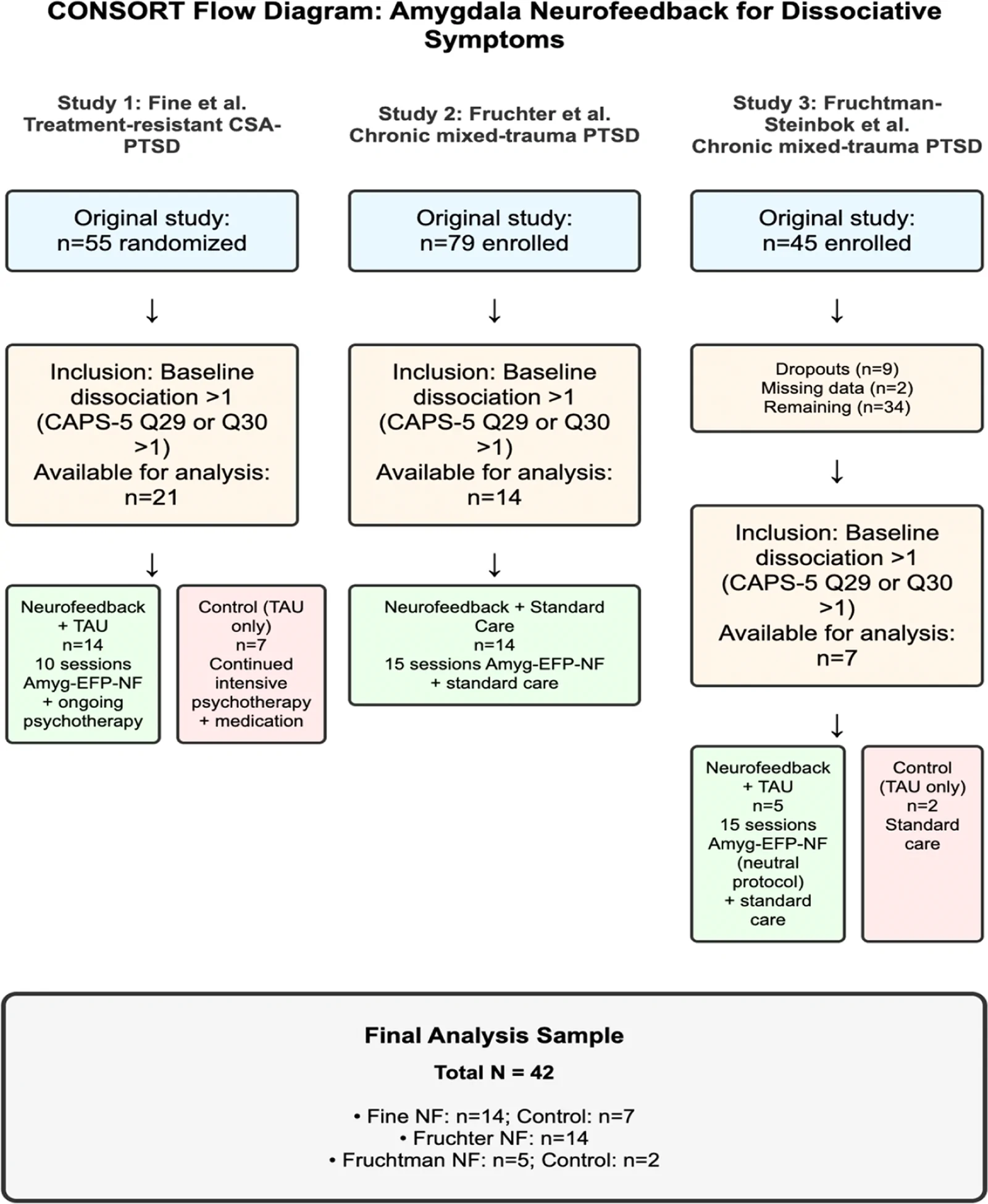
CONSORT flow diagram: amygdala neurofeedback for dissociative symptoms. Participant flow through the three independent studies examining amygdala-targeted neurofeedback for dissociative symptoms in PTSD. The flowchart shows screening, enrollment, allocation, and analysis phases for each study separately.

This analysis examined data from three independent studies that investigated amygdala-targeted neurofeedback in patients with PTSD. While these studies were conducted independently with different populations and protocols, all employed amygdala-targeted neurofeedback and assessed dissociative symptoms, enabling examination of consistency across research studies. The participant flow through all three studies is illustrated in Fig. 1. While the studies differed in design, population, and specific protocols, all employed self neuromodulation variants of amygdala-targeted EEG-fMRI pattern / fusion neurofeedback (Amyg-EFP-NF) and included assessment of dissociative symptoms using the Clinician-Administered PTSD Scale for DSM 5 (CAPS-5).

### 2.2. Study 1 (Fine et al.)

#### Design:

Comparison study examining amygdala neurofeedback plus treatment-as-usual versus treatment-as-usual alone (Fine et al., 2024).

#### Population:

Treatment-resistant survivors of childhood sexual abuse with PTSD who had been receiving ongoing therapy for more than one year without significant improvement in dissociative symptoms.

#### Intervention:

Ten sessions of Amyg-EFP-NF delivered over 9 weeks, with continued psychotherapy and medication management.

#### Control:

Continued intensive psychotherapy and medication management representing an active control condition.

#### Sample:

21 patients (14 neurofeedback, 7 control) who met inclusion criteria for clinically significant dissociative symptoms.

### 2.3. Study 2 (Fruchter et al.)

#### Design:

Single-arm multicenter study examining amygdala neurofeedback as an adjunctive intervention (Fruchter et al., 2024).

#### Population:

Individuals with chronic mixed-trauma PTSD recruited from five clinical trial sites.

#### Intervention:

Fifteen sessions of Amyg-EFP-NF delivered over 8 weeks, with concurrent standard care, additionally subjects were re-evaluated three months post treatment.

#### Sample:

14 patients with clinically significant baseline dissociative symptoms.

### 2.4. Study 3 (Fruchtman-Steinbok et al.)- pilot validation series

#### Design:

Comparison study examining amygdala neurofeedback plus treatment-as-usual versus treatment-as-usual alone (Fruchtman-Steinbok et al., 2021). Note: This represents a pilot validation series with limited statistical power due to small sample size.

#### Population:

Individuals with chronic mixed-trauma PTSD.

#### Intervention:

Fifteen sessions of Amyg-EFP-NF delivered over 13 weeks using neutral feedback interfaces (non-trauma-related audio and visual scenarios), with concurrent standard care.

#### Control:

Standard care alone.

#### Sample:

7 patients (5 neurofeedback, 2 control) with clinically significant baseline dissociative symptoms. Note that this analysis includes only participants who received the neutral neurofeedback protocol for consistency with the other studies.

### 2.5. Participant selection for current analysis

Participants were included in the current analysis if they demonstrated clinically significant baseline dissociative symptoms, defined as scores greater than 1 on either CAPS-5 question 29 (depersonalization) or question 30 (derealization). This threshold was selected to identify individuals for whom dissociative symptoms represented a clinically meaningful treatment target.

The final sample comprised 42 participants across the three studies: Fine study (14 neurofeedback + 7 control), Fruchter study (14 neurofeedback), and Fruchtman study (5 neurofeedback + 2 control). The rationale for this secondary analysis was to explore whether Amyg-EFP-NF specifically reduces dissociative symptoms in the subset of PTSD patients who present with this clinical feature.

### 2.6. Outcome measures

The primary outcome measure was change in dissociative symptoms as assessed by CAPS-5 questions 29 and 30, which evaluate depersonalization and derealization symptoms respectively (Weathers et al., 2013). These items are rated on a 5-point scale (0–4) based on frequency and intensity of symptoms. A total dissociation score was calculated as the sum of the two items (range 0–8).

Secondary outcome measures included individual depersonalization and derealization scores, clinical response rates defined as achieving subclinical levels (total dissociation score ≤1), and effect sizes for within-group and between-group comparisons.

CAPS-5 questions 29 and 30 represent the gold-standard clinical assessment for DSM-5-defined dissociative symptoms in PTSD, specifically targeting the depersonalization and derealization symptoms that define the dissociative subtype. These items have established psychometric properties and directly assess the core dissociative symptoms that were the focus of our intervention. While broader dissociation measures such as the Dissociative Experiences Scale (DES) assess trait dissociation across multiple contexts, CAPS-5 items specifically capture the state dissociative symptoms most relevant to PTSD treatment response. The clinical significance threshold (>1) was selected to identify individuals for whom dissociative symptoms represented a meaningful treatment target.

### 2.7. Statistical analysis

Study reporting followed CONSORT guidelines for randomized controlled trials where applicable (Schulz et al., 2010). Primary Analysis: Individual study results were analyzed using paired *t*-tests for within-group changes, with effect sizes calculated using Cohen’s d with 95 % confidence intervals (Cohen, 1988). Between-group comparisons used independent samples *t*-tests on change scores.

#### Secondary Analysis:

Mixed-effects models were employed to examine patterns across all studies while acknowledging protocol heterogeneity. These models included fixed effects for time, group (neurofeedback vs. control), and their interaction, with random effects for study to account for clustering.

#### Clinical Significance:

Response rate analysis examined the proportion of participants achieving subclinical dissociation levels (total score ≤1) in each group, with 95 % confidence intervals calculated using the Clopper-Pearson method.

Given the limited sample size in the Fruchtman study control group (*n* = 2), this data is presented as descriptive pilot information rather than definitive comparative evidence. Primary statistical comparisons focus on the larger Fine and Fruchter studies.

## 3. Results

### 3.1. Participant characteristics

Participant characteristics are summarized in Table 1. The participant characteristics reveal important heterogeneity across studies. The Fine study exclusively enrolled female survivors of childhood sexual abuse with early trauma onset, representing a treatment-resistant population. In contrast, the Fruchter and Fruchtman studies included more diverse samples with mixed trauma types and later average age at trauma onset. This heterogeneity reflects the independent nature of the studies and the different clinical populations they targeted.

### 3.2. Baseline and post-training dissociation scores

Baseline and post-training dissociation scores by study and group are presented in Table 2. All participants included in the analysis met criteria for clinically significant dissociative symptoms at baseline (CAPS-5 Q29/Q30 >1), as shown in Table 2.

### 3.3. Primary efficacy outcomes

**Fig. 2.**
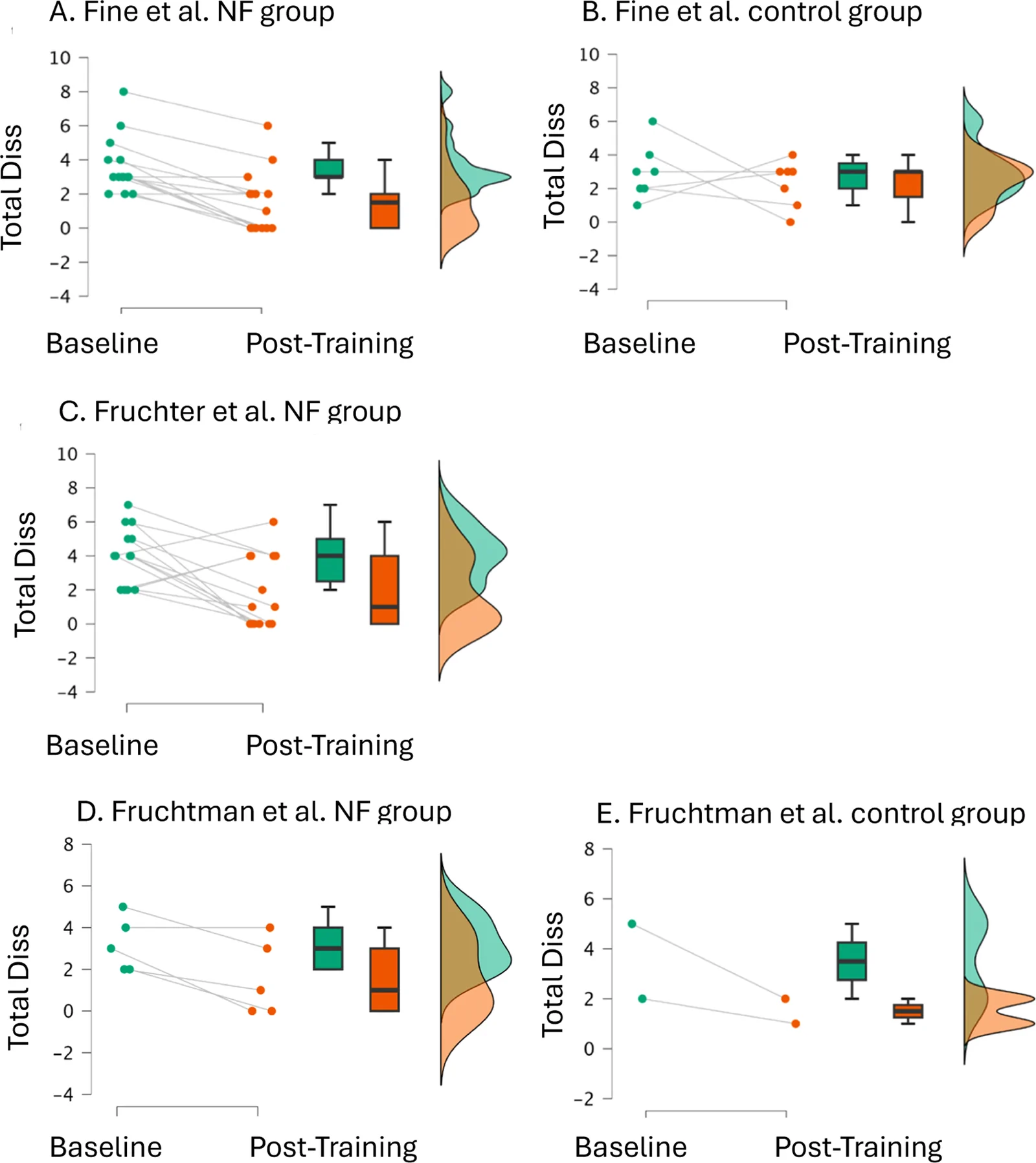
Individual participant trajectories and group changes by study. Individual participant trajectories and group-level changes in total dissociation scores across studies. Panel 2A shows the Fine neurofeedback group (*n* = 14) with individual participant lines connecting baseline to post-training scores, demonstrating consistent improvement across participants with large effect size. Panel 2B shows the Fine control group (*n* = 7) with minimal change. Panel 2C shows the Fruchter neurofeedback group (*n* = 14) with significant improvement. Panels 2D and 2E show the Fruchtman study neurofeedback (*n* = 5) and control (*n* = 2) groups, respectively. Individual trajectories are shown as connected lines (teal circles = baseline, orange circles = post-treatment). Box plots display group medians, quartiles, and ranges, while violin plots illustrate the probability density distributions of scores at each timepoint. The consistent downward trajectories in neurofeedback groups contrast with minimal changes in control conditions.

As shown in Fig. 2, individual participant trajectories in neurofeedback groups demonstrated consistent improvement patterns, with large significant effects across all neurofeedback studies contrasted with small non-significant effects in control groups receiving intensive treatment-as-usual for over one year.

### 3.4. Within-group effects: the core finding

The most consistent finding across all studies was large, statistically significant improvements in dissociative symptoms within neurofeedback groups. All three neurofeedback groups demonstrated substantial reductions in total dissociation scores: Fine NF (*d* = 1.82, t(13)=6.79, *p* <0.001), Fruchter NF (*d* = 0.85, t(13)=3.18, *p* = 0.007). The Fruchtman study showed similar patterns but did not reach statistical significance due to small sample size (*n* = 5).

In contrast, control groups showed minimal improvements that did not reach statistical significance: Fine Control (*d* = 0.28, t(6)=0.74, *p* = 0.489) and Fruchtman Control (*d* = 1.33, t(1)=1.41, *p* = 0.387, underpowered with *n* = 2). The Fruchtman study, while showing consistent patterns, had insufficient power for definitive statistical conclusions due to small sample sizes, particularly in the control group (*n* = 2).

These findings are particularly notable given that the Fine study control group represented an active control condition, with participants receiving intensive psychotherapy and medication management for over one year without significant improvement in dissociative symptoms prior to study entry.

### 3.5. Between-group comparisons

Direct between-group comparisons were limited by small control group sizes but revealed consistent patterns favoring neurofeedback. The Fine study showed a between-group effect size of *d* = 0.68, representing a medium-to-large effect favoring neurofeedback. When combining Fine and Fruchtman studies, the between-group effect size was *d* = 0.41, representing a small-to-medium effect favoring neurofeedback.

### 3.6. Subgroup analysis by dissociation type

**Fig. 3.**
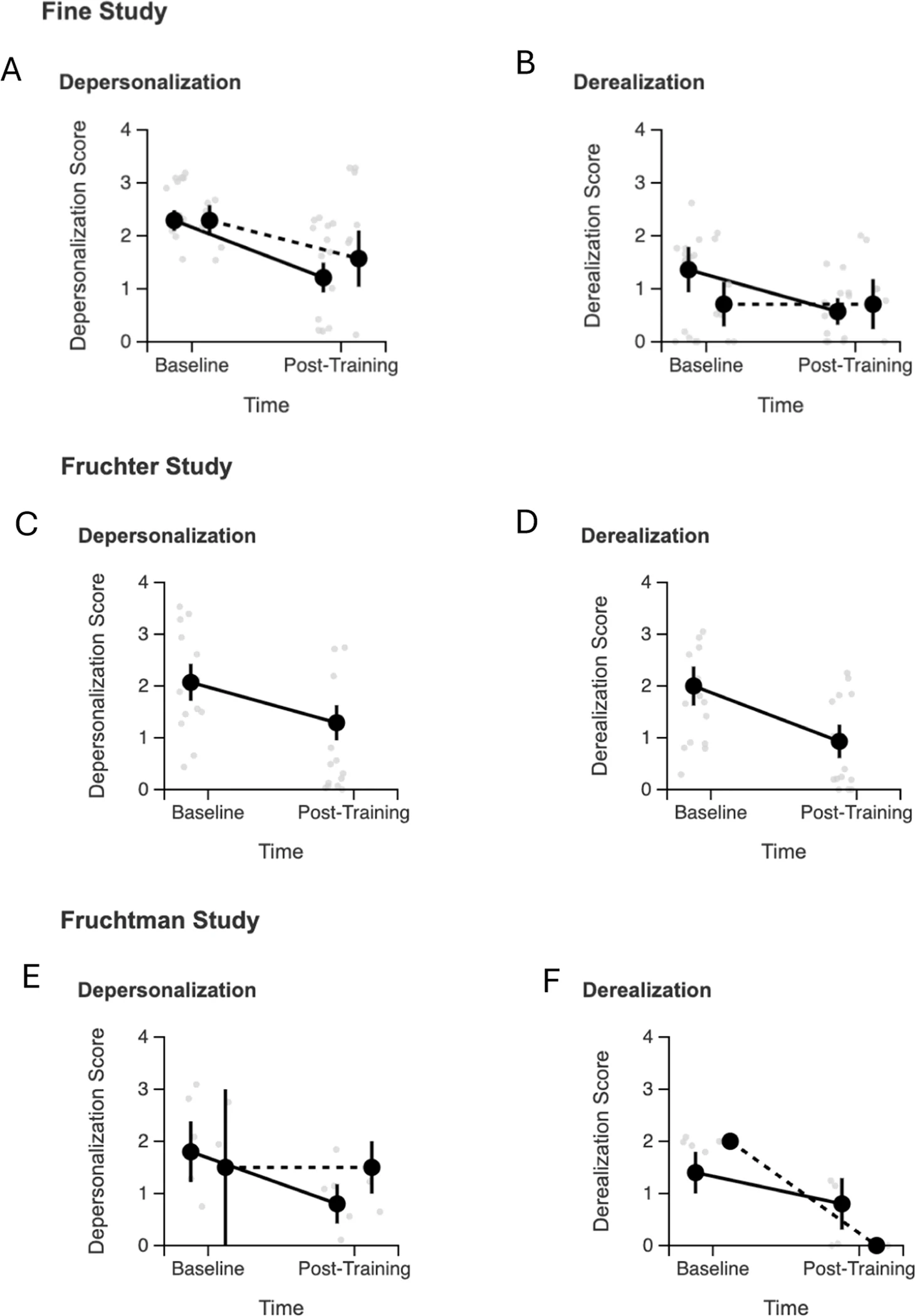
Depersonalization and derealization symptom changes by study. Depersonalization and derealization symptom changes by study and treatment group. Individual participant data points (gray circles) show raw scores at baseline and post-training. Black circles represent group means with standard errors (error bars). Solid lines connect neurofeedback group means; dashed lines connect control group means where available. Fine study (panel A-B) included both neurofeedback (*n* = 14) and control (*n* = 7) groups. Fruchter study (panel C-D) was single-arm with neurofeedback only (*n* = 14). Fruchtman study (panel E-F) included neurofeedback (*n* = 5) and control (*n* = 2) groups. Both depersonalization and derealization symptoms showed consistent improvements in neurofeedback groups across studies, with larger effect sizes than control conditions.

As shown in Fig. 3, both depersonalization and derealization symptoms demonstrated significant improvements in neurofeedback groups. For depersonalization symptoms, the Fine neurofeedback group achieved a large effect size (*d* = 1.46, t(13)=5.47, *p* < 0.001), while the Fruchter group showed a medium effect (*d* = 0.61, t(13)=2.28, *p* = 0.040). For derealization symptoms, significant improvements were observed in the Fine neurofeedback group (*d* = 0.72, t(13)=2.69, *p* = 0.019) and the Fruchter group (*d* = 0.94, t(13)=3.51, *p* = 0.004). The Fruchtman study showed similar non-significant patterns for both symptom types. The pattern of results suggests that neurofeedback was effective for both types of dissociative symptoms, with some variation in effect sizes across studies potentially reflecting differences in baseline severity and population characteristics.

### 3.7. Mixed-effects model results

**Fig. 4.**
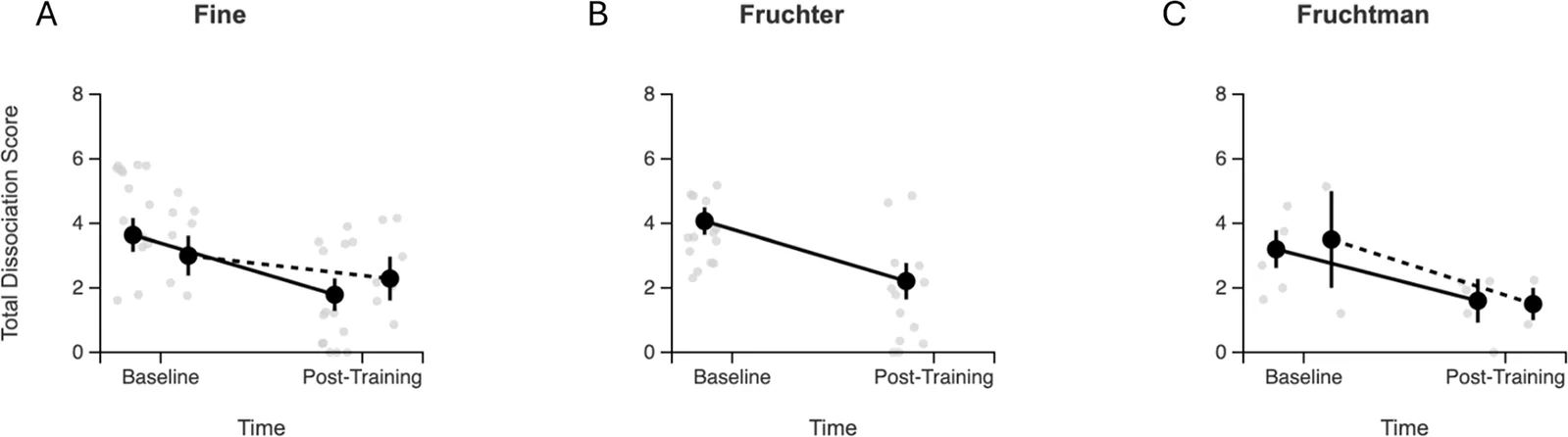
Longitudinal changes in total dissociation scores by study. Longitudinal changes in total dissociation scores by study and treatment group. Individual participant data points (gray circles) show raw scores at baseline and post-training with slight horizontal jittering for visibility. Black circles represent group means with standard errors (error bars). Solid lines connect neurofeedback group means; dashed lines connect control group means where available. Fine study (A) included both neurofeedback (*n* = 14) and control (*n* = 7) groups. Fruchter study (B) was single-arm with neurofeedback only (*n* = 14). Fruchtman study (C) included neurofeedback (*n* = 5) and control (*n* = 2) groups. All neurofeedback groups demonstrated substantial reductions in total dissociation scores, with steeper improvement slopes compared to control conditions where available.

Mixed-effects models were employed to examine overall patterns while accounting for study-level clustering. As illustrated in Fig. 4, the analysis revealed significant main effects of time across all studies, indicating overall improvement in dissociative symptoms. In the combined studies analysis including all three studies, there was a significant time effect (F(1,32)=22.03, *p* < 0.001) with a non-significant Group × Time interaction (F(1,32)=0.72, *p* = 0.404). For the Fine study’s controlled comparison specifically, there was a significant time effect (F (1,19)=24.76, *p* < 0.001) with a non-significant Group × Time interaction (F(1,19)=1.36, *p* = 0.259). While the Group × Time interactions did not reach statistical significance, the pattern consistently favored neurofeedback groups with steeper improvement slopes. The non-significant interactions likely reflect limited statistical power due to small control group sizes and heterogeneity in control conditions across studies.

### 3.8. Clinical response analysis

Clinical response was defined as achieving subclinical levels of dissociative symptoms (total dissociation score ≤1) at post-treatment assessment. This threshold was selected based on the clinical significance of moving from symptomatic to subclinical levels.

The response rate analysis, summarized in Table 3, revealed consistently higher rates of clinical response in neurofeedback groups compared to controls. Over half of neurofeedback participants achieved subclinical dissociation levels, compared to approximately 30 % of controls (acknowledging the limited control group data). While these differences did not reach statistical significance due to sample size limitations, the consistent pattern and associated number needed to treat values (4–4.7) suggest potentially meaningful clinical effects.

### 3.9. Follow-up analysis

**Fig. 5.**
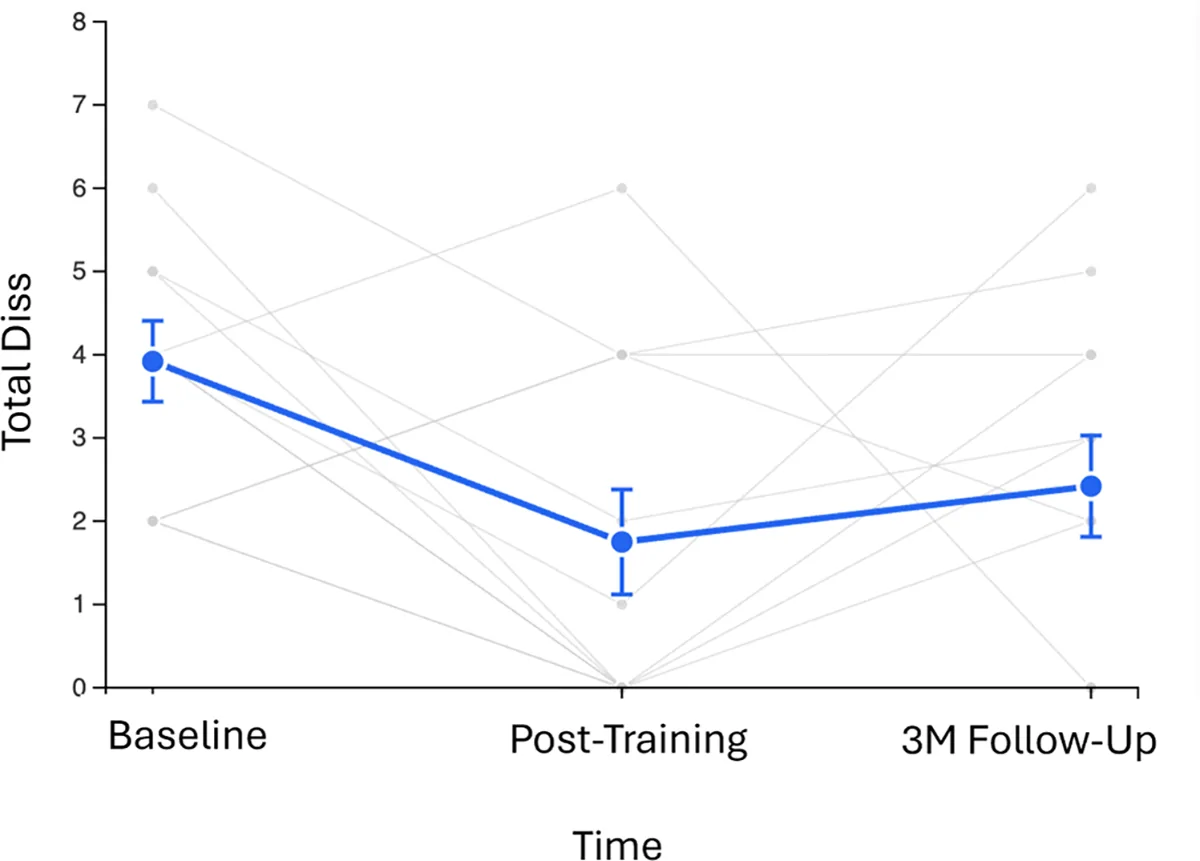
Follow-up analysis. Three-month follow-up analysis showing durability of amygdala-targeted neurofeedback effects (*n* = 12). Individual participant trajectories (gray lines) and group means with standard error bars (blue line) demonstrate sustained improvement compared to baseline (*d* = 0.79, t(11)=1.96, *p* = 0.07) despite modest symptom return from post-treatment. Six of 12 participants (50 %) showed continued improvement or maintenance from post-treatment, and 4 of 12 participants (33 %) maintained clinical response levels at follow-up.

Preliminary 3-month follow-up data from the Fruchter study (*n* = 12) were analyzed to provide initial insights into treatment durability (Fig. 5). Participants maintained improvements in total dissociation scores compared to baseline (baseline: *M* = 3.92, SD=1.68; 3-month follow-up: *M* = 2.42, SD=2.11; *d* = 0.79, t(11)=1.96, *p* = 0.07), indicating durability of treatment effects. While there was a modest increase from post-treatment (*M* = 1.75, SD=2.18) to 3-month follow-up (*d*=−0.31), scores remained numerically below baseline levels, suggesting sustained clinical benefit.

Individual trajectory analysis revealed that 6 of 12 participants (50 %) maintained gains from post-training and 3 of 12 participants (33 %) maintained clinically meaningful improvement at follow-up, with 4 participants showing some symptoms return but remaining below baseline levels. The sustained medium-large effect size (*d* = 0.79) from baseline to follow-up supports the durability of amygdala-targeted neurofeedback benefits for dissociative symptoms.

### 3.10. Specificity analysis

To examine whether dissociation improvements represented specific effects rather than general PTSD symptom reduction, we analyzed correlations between change scores in dissociation symptoms and changes in other PTSD symptom clusters across all neurofeedback participants (*N* = 33).

For total dissociation score changes (Q29+Q30), correlations with other CAPS-5 domains were negligible: total CAPS-5 score (*r* = −0.07), Cluster B intrusion symptoms (*r* = −0.13), Cluster C avoidance symptoms (*r* = −0.13), Cluster D negative alterations in cognition and mood (*r* = 0.03), and Cluster E alterations in arousal and reactivity (*r* = −0.06).

Analysis of individual dissociation components revealed similar patterns. Depersonalization improvements (Q29) showed weak correlations with other symptoms domains: total CAPS-5 (*r* = 0.07), Cluster B (*r* = −0.05), Cluster C (*r* = −0.07), Cluster D (*r* = 0.15), and Cluster E (*r* = 0.09). Derealization improvements (Q30) also demonstrated independence from other PTSD symptoms: total CAPS-5 (*r* = −0.17), Cluster B (*r* = −0.11), Cluster C (*r* = −0.02), Cluster D (*r* = −0.13), and Cluster E (*r* = −0.20).

All correlations fell well below conventional thresholds for meaningful associations (*r* < 0.30). These findings indicate that both depersonalization and derealization improvements occurred largely independently of changes in other PTSD symptom domains, supporting specificity of amygdala-targeted neurofeedback for dissociative symptoms rather than general therapeutic effects,

### 3.11. Effect size summary

The effect size summary (Table 4) demonstrates a clear pattern of large effects in neurofeedback groups contrasted with minimal effects in control groups across all measures of dissociative symptoms.

## 4. Discussion

### 4.1. Primary findings: convergent evidence pattern

This analysis provides convergent evidence from two primary independent studies and one pilot validation series supporting the potential therapeutic benefit of amygdala-targeted neurofeedback for dissociative symptoms in PTSD. The most compelling finding is the consistent pattern of large within-group effect sizes across all neurofeedback conditions (*d* = 0.85–1.82), accompanied by minimal improvements in control groups where available.

The specificity analysis provides compelling evidence that observed improvements were not simply reflective of general PTSD symptom reduction. The consistently weak correlations between dissociation and other symptom clusters suggest that amygdala neurofeedback may target neurobiological mechanisms specifically underlying dissociative experiences, consistent with theoretical models proposing distinct neural pathways for dissociative versus hyperarousal PTSD presentations. This finding is particularly notable given established efficacy of amygdala-targeted neurofeedback for PTSD broadly, suggesting that the dissociation-specific effects represent a distinct therapeutic mechanism rather than a secondary consequence of general PTSD improvement.

The clinical context of these findings enhances their significance. In the Fine study, the control group consisted of individuals receiving intensive ongoing psychotherapy and medication management for over one year without meaningful improvement in dissociative symptoms (Fine et al., 2024). The large effect size (*d* = 1.82) observed in the neurofeedback group represents improvement beyond what was achieved through prolonged intensive treatment-as-usual, suggesting that neurofeedback may offer unique therapeutic benefits for this challenging symptom domain.

The independent replication of beneficial effects across different research studies, populations, and treatment contexts strengthens confidence in these preliminary findings (Fine et al., 2024; Fruchter et al., 2024; Fruchtman-Steinbok et al., 2021). The Fruchter study, conducted independently with a different population of mixed-trauma survivors, demonstrated similar patterns of improvement (*d* = 0.85), while the Fruchtman study, despite its small sample size, showed consistent large effects (*d* = 1.08).

Clinical response rate analysis revealed that over half of neurofeedback participants achieved subclinical levels of dissociative symptoms, compared to approximately 30 % of controls. While statistical significance was limited by sample sizes, the associated number needed to treat values (4–4.7) suggest clinically meaningful effects that might be more definitively established in larger samples.

### 4.2. Methodological considerations and study heterogeneity

The three studies included in this analysis represent independent investigations that, while differing substantially in methodological approaches, populations, and control conditions, provide an opportunity to examine consistency of findings across research studies. These differences both strengthen and limit the interpretability of the findings.

The consistency of beneficial effects despite these methodological differences suggests that the core intervention approach may be robust across reasonable variations in implementation. However, the heterogeneity limits direct statistical pooling and requires careful interpretation of combined analyses.

Protocol Differences included variation in treatment number (10 vs. 15 sessions), total duration (9 vs. 8–13 weeks), and feedback interface design (Fine et al., 2024; Fruchter et al., 2024; Fruchtman-Steinbok et al., 2021). Despite these protocol differences, all three studies demonstrated beneficial effects, suggesting that the core intervention approach may be robust across reasonable variations in implementation.

Population Differences were substantial across studies. The Fine study exclusively enrolled female survivors of childhood sexual abuse representing a treatment-resistant population, while the Fruchter and Fruchtman studies included more diverse samples with mixed trauma types and later average age at trauma onset. The consistency of beneficial effects across these different populations suggests that amygdala-targeted neurofeedback may have broad applicability for dissociative symptoms regardless of specific trauma history.

Control Condition Differences represent both a strength and limitation of this analysis. The Fine study employed an active control condition with continued intensive therapy, providing a stringent test of neurofeedback efficacy beyond standard care (Fine et al., 2024). The Fruchtman study used standard care as a control (Fruchtman-Steinbok et al., 2021), while the Fruchter study was single-arm (Fruchter et al., 2024). This heterogeneity limits the interpretability of pooled analyses but provides complementary evidence across different comparison conditions.

### 4.3. Statistical interpretation: pattern vs. power

The non-significant Group × Time interactions in mixed-effects models require careful interpretation. These null findings likely reflect statistical power limitations due to small control group sizes (*n* = 7, *n* = 2) rather than absence of treatment effects. The consistent pattern of results across all analyses favored neurofeedback, with effect sizes suggesting meaningful clinical differences even when statistical significance was not achieved.

This analysis prioritizes pattern consistency over statistical power from any single comparison. The replication of large within-group effects across three independent studies provides stronger evidence than would be available from any single study alone, even with larger sample sizes. This approach is particularly appropriate given the early stage of this research field and the practical challenges of conducting large-scale neurofeedback trials.

### 4.4. Clinical translation implications

These findings have several important implications for clinical practice and future research. The evidence suggests that amygdala-targeted neurofeedback may be particularly beneficial for PTSD patients presenting with clinically significant dissociative symptoms, a population that often shows limited response to standard trauma-focused treatments.

The scalable nature of EEG-based neurofeedback technology makes this intervention potentially more accessible than fMRI-based approaches, while the EEG-fMRI fusion methodology enables targeting precision that approaches that of real-time fMRI (Keynan et al., 2016). This combination of accessibility and precision positions amygdala-targeted neurofeedback as a promising adjunctive intervention for dissociative PTSD.

The evidence for benefit beyond intensive treatment-as-usual suggests that neurofeedback may address neurobiological mechanisms that are not effectively targeted by traditional psychotherapy and medication approaches. This finding is particularly relevant for treatment-resistant populations who have exhausted standard therapeutic options.

### 4.5. Neurobiological mechanisms and theoretical implications

The consistent beneficial effects observed across studies provide indirect support for theoretical models proposing that dissociative symptoms of depersonalization and derealization arise, at least in part, from dysregulated amygdala-mediated networks (Lanius et al., 2010; Menon, 2015; Nicholson et al., 2017). The effectiveness of amygdala-targeted interventions suggests that this brain region plays a central role in the neural mechanisms underlying dissociative experiences.

The improvements observed in both depersonalization and derealization symptoms support models proposing shared underlying mechanisms for different types of dissociative experiences (Sierra and Berrios, 1998; Simeon and Abugel, 2006). The amygdala’s role in salience processing, interoception, and self-referential awareness positions it as a plausible common pathway through which different dissociative symptoms might emerge and be therapeutically modified (Cunningham and Brosch, 2012; Menon, 2015).

The pattern of effects also supports network-based models of dissociation over simple regional activation theories (Menon, 2015; Chiba et al., 2021). Rather than simply “turning down” amygdala activity, neurofeedback training may help restore flexible regulatory patterns that enable more adaptive responses to internal and external stimuli. This interpretation is consistent with contemporary theories emphasizing regulatory flexibility over static activation levels (Ros et al., 2013).

### 4.6. Study strengths and limitations

Strengths of this analysis include the demonstration of consistency across separate studies and populations (Fine et al., 2024; Fruchter et al., 2024; Fruchtman-Steinbok et al., 2021). The inclusion of a stringent active control condition in the Fine study provides evidence for effects beyond intensive standard care (Fine et al., 2024). The range of trauma populations and treatment contexts enhances the generalizability of findings. Large effect sizes with confidence intervals provide clinically meaningful estimates of treatment benefit (Cohen, 1988).

Limitations include protocol heterogeneity that limits direct pooling of results and small control group sizes that limit statistical power for between-group comparisons. The different control conditions across studies make direct comparisons problematic. Non-significant interactions may reflect power limitations rather than absence of effects. This represents a secondary analysis of existing datasets rather than a prospectively planned investigation. The Fruchtman study control group was extremely small (*n* = 2), limiting its contribution to between-group analyses.

### 4.7. Future research directions

Immediate priorities for future research include larger randomized controlled trials with adequate statistical power to detect between-group differences. Placebo-controlled designs using sham neurofeedback conditions would help establish specific efficacy beyond non-specific factors. Standardized protocols and assessment schedules across sites would enable more definitive pooled analyses.

Mechanistic studies should investigate the neuroimaging correlates of treatment response to validate proposed mechanisms of action. Identification of predictive biomarkers could enable treatment selection and personalization. Dose-response optimization studies could determine optimal session numbers and scheduling. Investigation of durability through larger-scale longer-term follow-up studies is needed to establish the persistence of benefits across populations and time periods.

Clinical implementation research should examine real-world effectiveness in diverse clinical settings, cost-effectiveness compared to standard treatments, and optimal integration with existing therapeutic approaches. Training and certification protocols for clinicians would facilitate widespread implementation if efficacy were established.

Future studies should incorporate dedicated dissociation measures such as the Dissociative Experiences Scale (DES) or Multidimensional Inventory of Dissociation (MID) to complement CAPS-5 items and provide comprehensive dissociative symptom assessment.

### 4.8. Clinical implications and recommendations

These preliminary findings from separate studies suggest potential therapeutic benefit and support continued investigation of amygdala-targeted neurofeedback as a potentially valuable addition to the therapeutic armamentarium for dissociative PTSD (Fine et al., 2024; Fruchter et al., 2024; Fruchtman-Steinbok et al., 2021). The convergent evidence across independent studies, combined with large effect sizes and clinical response rates, warrants progression to larger definitive trials.

Clinicians working with treatment-resistant PTSD patients, particularly those with prominent dissociative symptoms, may consider referring patients for neurofeedback evaluation as part of a comprehensive treatment approach. However, these interventions should be considered investigational until larger controlled trials establish definitive efficacy.

The individual differences observed in treatment response highlight the importance of personalized approaches to dissociative PTSD. Future research should focus on identifying predictive factors that can guide treatment selection and optimize outcomes for individual patients.

### 4.9. Conclusion

This analysis provides convergent preliminary evidence from three independent studies that amygdala-targeted neurofeedback may offer therapeutic benefit for dissociative symptoms in PTSD. The consistent pattern of large within-group effects across different populations and protocols (*d* = 0.85–1.82), combined with higher clinical response rates in neurofeedback groups, supports the therapeutic potential of this intervention.

The evidence is particularly compelling given that beneficial effects were observed beyond intensive treatment-as-usual in treatment-resistant populations. The independent replication across research studies strengthens confidence in these preliminary findings and suggests that the intervention may have broad applicability for dissociative PTSD.

While protocol heterogeneity and small control groups limit definitive between-group conclusions, the convergent evidence pattern warrants progression to larger randomized controlled trials with standardized protocols. Such studies are necessary to establish the efficacy of this promising, scalable intervention and to determine its optimal role in the treatment of dissociative PTSD.

The neurobiological plausibility of amygdala-targeted interventions for dissociative symptoms, combined with the preliminary evidence presented here, suggests that this approach may address fundamental mechanisms underlying dissociative experiences that are not effectively targeted by traditional therapeutic approaches. If confirmed in larger trials, amygdala-targeted neurofeedback could represent an important advance in the treatment of one of the most challenging presentations of PTSD.

## Figures and Tables

**Table 1:** Participant characteristics by study and group.

Characteristic	Fine NF (*n* = 14)	Fine Control (*n* = 7)	Fruchter NF (*n* = 14)	Fruchtman NF (*n* = 5)	Fruchtman Control (*n* = 2)	Total (*n* = 42)

**Age, M(SD)**	35.7(11.6)	41.3(8.0)	38.9(12.3)	40.4(8.5)	25.5(4.9)	37.4(11.3)
**Female, n( %)**	14(100.0)	7(100.0)	9(64.3)	3(60.0)	1(50.0)	34(81.0)
**Age at trauma onset, M(SD)**	7.7(2.9)	8.4(4.0)	31.0(11.2)	36.3(9.1)	21.3(3.3)	20.1(14.5)
**Trauma Type, n( %)**						
Combat/Military	0(0.0)	0(0.0)	6(42.9)	2(40.0)	1(50.0)	9(21.4)
Childhood sexual abuse	14(100.0)	7(100.0)	0(0.0)	0(0.0)	0(0.0)	21(50.0)
Sexual trauma	0(0.0)	0(0.0)	1(7.1)	2(40.0)	0(0.0)	2(4.8)
Other	0(0.0)	0(0.0)	7(50.0)	1(20.0)	1(50.0)	10(23.8)
**Baseline CAPS-5 Total**	39.4(9.9)	50.6(11.0)	45.8(6.6)	41.8(6.0)	42.0(1.4)	43.0(9.2)

**Table 2:** Baseline and post-training dissociation scores by study and group.

Study/Group	Measure	Baseline M(SD)	Post-Training M (SD)	Effect Size d [95 % CI]

**Fine Study**				
Neurofeedback (*n* = 14)	Total Dissociation	3.64 (1.95)	1.79 (1.89)	1.82* [0.94, 2.67]
	Depersonalization	2.29 (0.73)	1.21 (1.05)	1.46* [0.69, 2.21]
	Derealization	1.36 (1.60)	0.57 (0.94)	0.72* [0.12, 1.30]
Control (*n* = 7)	Total Dissociation	3.00 (1.63)	2.29 (1.80)	0.28 [−0.49, 1.02]
	Depersonalization	2.29 (0.76)	1.57 (1.40)	0.66 [−0.19, 1.46]
	Derealization	0.71 (1.11)	0.71 (1.25)	0.59 [−0.24, 1.37]
**Fruchter Study**				
Neurofeedback (*n* = 14)	Total Dissociation	4.07 (1.59)	2.21 (2.12)	0.85** [0.22, 1.45]
	Depersonalization	2.07 (1.33)	1.29 (1.27)	0.61* [0.03, 1.17]
	Derealization	2.00 (1.41)	0.93 (1.21)	0.94** [0.29, 1.56]
**Fruchtman Study**				
Neurofeedback (*n* = 5)	Total Dissociation	3.20 (1.30)	1.60 (1.52)	1.08* [0.19, 1.97]
	Depersonalization	1.80 (1.30)	0.80 (0.84)	0.91* [0.06, 1.76]
	Derealization	1.40 (0.89)	0.80 (1.10)	0.60 [−0.16, 1.36]
Control (*n* = 2)	Total Dissociation	3.50 (2.12)	1.50 (0.71)	1.33 [−0.45, 3.11]
	Depersonalization	1.50 (2.12)	1.50 (0.71)	0.00 [−1.41, 1.41]
	Derealization	2.00 (0.00)	0.00 (0.00)	-

* *p* < 0.05,.

** *p* < 0.01.

**Table 3:** Clinical response analysis (total dissociation score ≤1).

Group	Responders/Total	Response Rate	95 % CI

Fine Control	2/7	28.6 %	[3.7 %, 71.0 %]
Fine NF	7/14	50.0 %	[23.0 %, 77.0 %]
Fruchter NF	8/14	57.1 %	[28.9 %, 82.3 %]
Fruchtman Control	1/2	50.0 %	[1.3 %, 98.7 %]
Fruchtman NF	3/5	60.0 %	[14.7 %, 94.7 %]
**Combined NF**	**18/33**	**54.5 %**	**[36.4 %, 71.9 %]**

**Table 4:** Comprehensive effect size comparison.

Group	N	Total Diss	95 % CI	DP	95 % CI	DR	95 % CI

**Fine NF**	14	1.82***	0.94–2.67	1.46***	0.69–2.21	0.72*	0.12–1.30
**Fine Control**	7	0.28 ns	−0.49–1.02	0.66 ns	−0.19–1.46	0.59 ns	−0.24–1.37
**Fruchter NF**	14	0.85**	0.22–1.45	0.61*	0.03–1.17	0.94**	0.29–1.56
**Fruchtman NF**	5	1.08*	0.19–1.97	0.91*	0.06–1.76	0.60 ns	−0.16–1.36

* *p* < 0.05,.

** *p* < 0.01,.

*** *p* < 0.001, ns=non-significant, DP = Depersonalization; DR = Derealization; Total Diss = Total Dissociation Score.

## Data Availability

The data that support the findings of this study are available from the corresponding author upon reasonable request, subject to appropriate ethical approvals and data sharing agreements.
